# Supplementary material for: Endothelin-1 signaling maintains glial progenitor proliferation in the postnatal subventricular zone
Source: Nat Commun. 2020 May 1;11:2138. doi: 10.1038/s41467-020-16028-8 (PMC7195367; doi:10.1038/s41467-020-16028-8)
Supplement: Supplementary file 1 — Supplementary Information [file 41467_2020_16028_MOESM1_ESM.pdf]

Supplementary Information for:

Endothelin-1 signaling maintains glial progenitor proliferation in the postnatal subventricular zone

Katrina L Adams[1], Giulia Riparini[2], Payal Banerjee[3], Marjolein Breur[4,5], Marianna Bugiani[4,5], and Vittorio Gallo[1*].

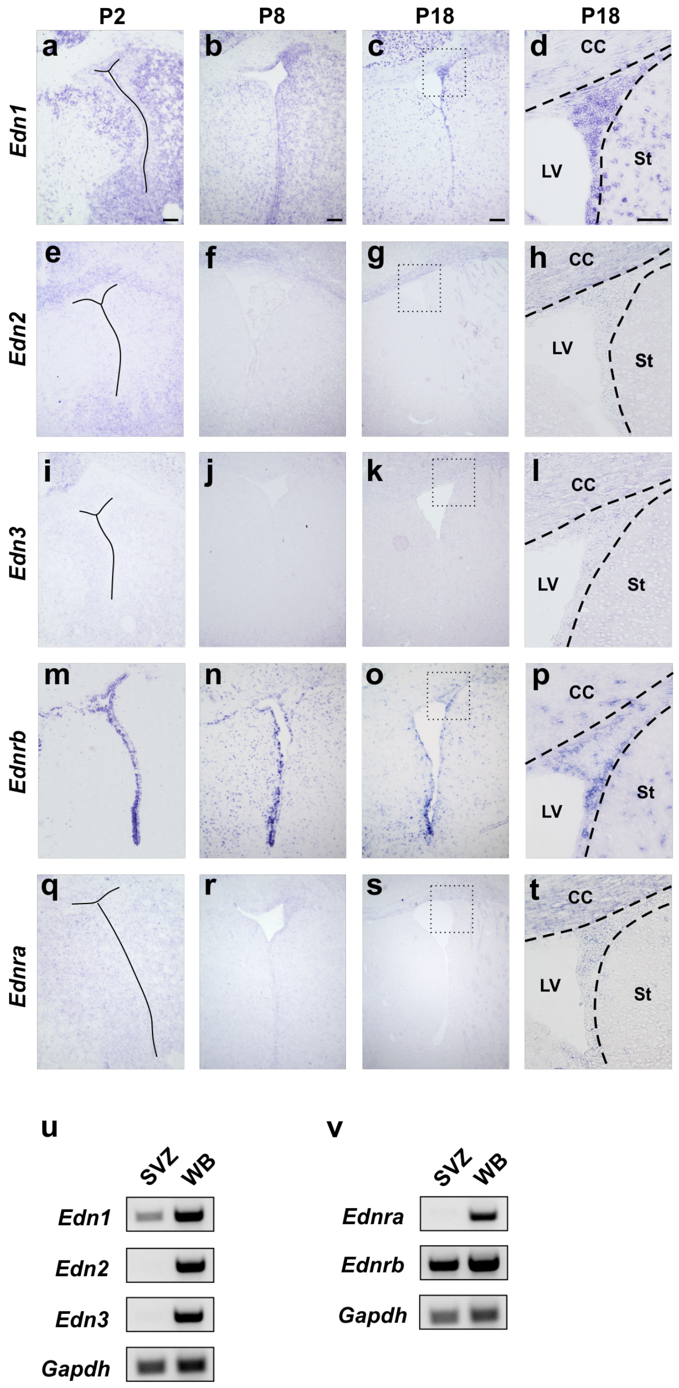

Supplementary Figure 1. Expression profile of Endothelin pathway proteins in the postnatal mouse brain.

(**a-d**) In situs for *Edn1* expression in WT mouse brain at postnatal day 2 (P2), P8, and P18. Images are focused on the right lateral ventricle (LV). Boxed region in c is magnified in d. Position of LV is drawn in a. Dorsolateral SVZ region is outlined in d. CC = corpus callosum. St = striatum. (**e-h**)

In situs for *Edn2* expression in WT mouse brain at P2, P8, and P18. Boxed region in g is magnified in h. (**i-l**) In situs for *Edn3* expression in WT mouse brain at P2, P8, and P18. Boxed region in k is magnified in l. (**m-p**) In situs for *Ednrb* expression in WT mouse brain at P2, P8, and P18. Boxed region in o is magnified in p. (**q-t**) In situs for *Ednra* expression in WT mouse brain at P2, P8, and P18. Boxed region in s is magnified in t. In situ images are representative of 3 WT mice. (**u**) RT-PCR for *Edn1*, *Edn2*, *Edn3*, and *Gapdh* using P7 SVZ or whole-brain (WB) derived cDNA. (**v**) RT-PCR for *Ednrb*, *Ednra*, and *Gapdh* using P7 SVZ or WB-derived cDNA. RT-PCR was replicated twice. Scale bars = 100μm for P2, P8, and P18 (c, g, k, o, s). Scale bar = 50μm for P18 (d, h, l, p, t). Source data are provided as a Source Data file.

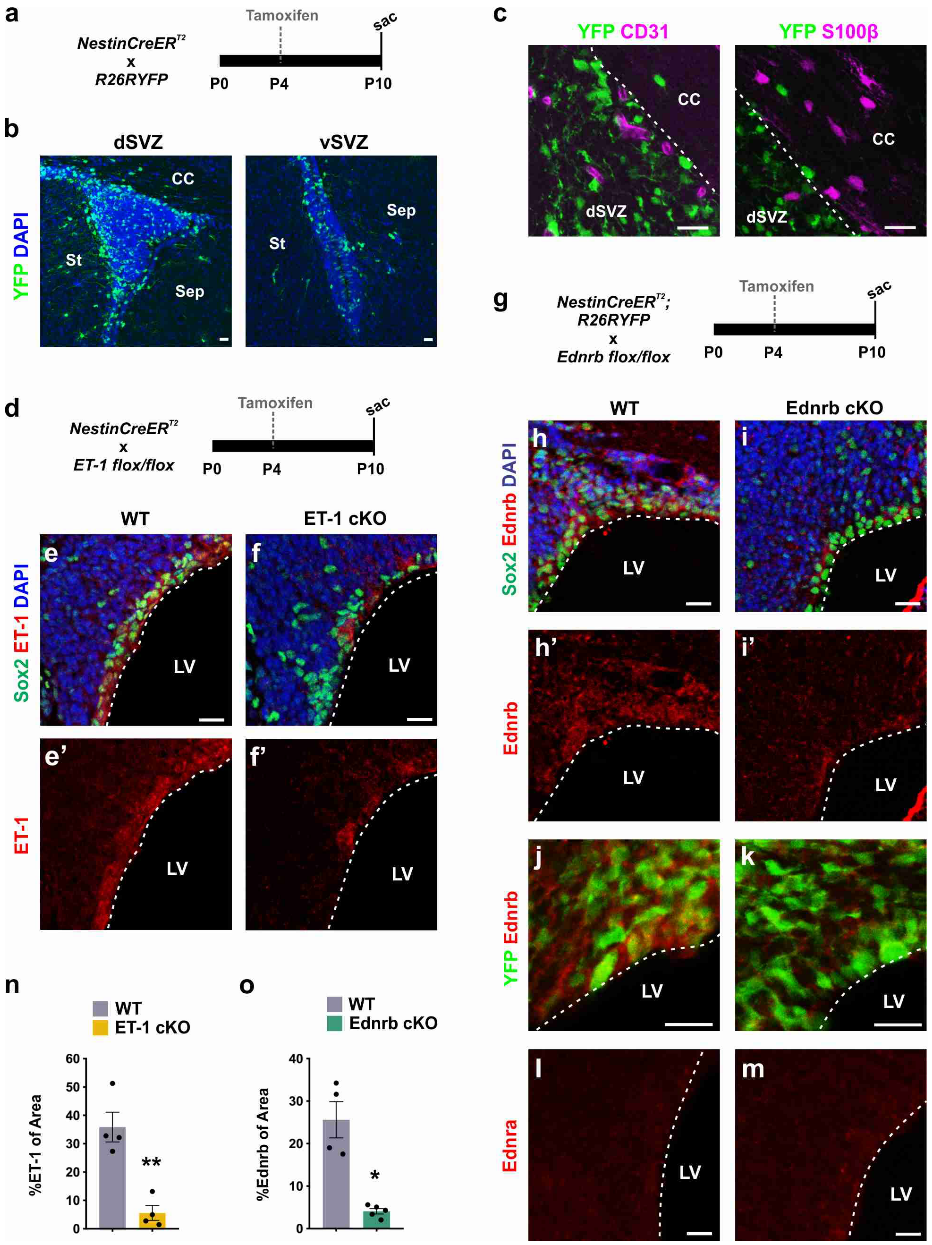

Supplementary Figure 2. Method and confirmation of ET-1 and Ednrb knockdown in the postnatal SVZ.

(**a**) Experimental strategy for images in b and c. (**b**) Coronal sections of P10 *Nestin::CreER*^{T2}*; R26RYFP* mouse dorsal and ventral SVZ. CC = corpus callosum, dSVZ = dorsal subventricular zone, Sep = septum, St = striatum, vSVZ = ventral subventricular zone. Images are representative of 3 *Nestin::CreER*^{T2}*; R26RYFP* mice. (**c**) YFP+ recombined cells in *Nestin::CreER*^{T2}*; R26RYFP* mice do not express markers of endothelial cells (CD31) or white matter astrocytes (S100β). Images are representative of 3 *Nestin::CreER*^{T2}*; R26RYFP* mice. (**d**) Experimental strategy for

ET-1 ablation in the developing postnatal SVZ. (**e-f**) ET-1 protein is reduced in Sox2+ progenitors lining the lateral ventricle (LV) of P10 ET-1 cKO mice (f), compared to WT controls (e). e' and f' show single channel images for ET-1 staining. Images are representative of 4 WT and ET-1 cKO mice. (**g**) Experimental strategy for Ednrb ablation in the developing postnatal SVZ. (**h-i**) Ednrb protein in reduced in Sox2+ progenitors lining the LV of P10 Ednrb cKO mice (i), compared to WT controls (h). h' and i' show single channel images for Ednrb staining. (**j-k**) Ednrb protein is reduced in YFP+ recombined cells in the SVZ of Ednrb cKO mice (k), compared to WT controls (j). (**l-m**) Ednra protein is not expressed at detectible levels within the SVZ of WT (l) and Ednrb cKO mice (m). Images are representative of 4 WT and 5 Ednrb cKO mice. (**n**) Quantification of percentage of ET-1+ pixels per area in the SVZ in WT and ET-1 cKO mice at P10 (n=4 mice both groups). **p-value = 0.0052 (Welch's t test). (**o**) Quantification of percentage of Ednrb+ pixels per area in the SVZ in WT and Ednrb cKO mice at P10 (n=4 WT, 5 Ednrb cKO mice). *p-value = 0.0140 (Welch's t test). All scale bars = 25μm. LV = lateral ventricle. Data are presented as mean values +/- SEM. Source data are provided as a Source Data file.

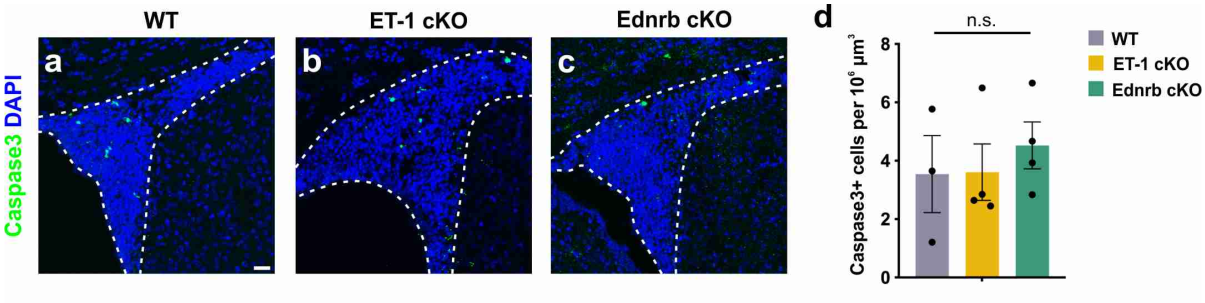

Supplementary Figure 3. Ablation of ET-1 or Ednrb in the SVZ does not affect cellular apoptosis.

(**a-c**) Coronal sections of the dorsal SVZ (outlined in white) in WT (a), ET-1 cKO (b), and Ednrb cKO (c) mice at P10. Scale bars = 25µm. (**d**) Quantification of the number of Caspase3+ cells in the SVZ, normalized to area (n=3 WT, 4 ET-1 cKO, 4 Ednrb cKO mice). n.s. = not significant (One-way ANOVA with Tukey's multiple comparisons test). Data are presented as mean values +/- SEM. Source data are provided as a Source Data file.

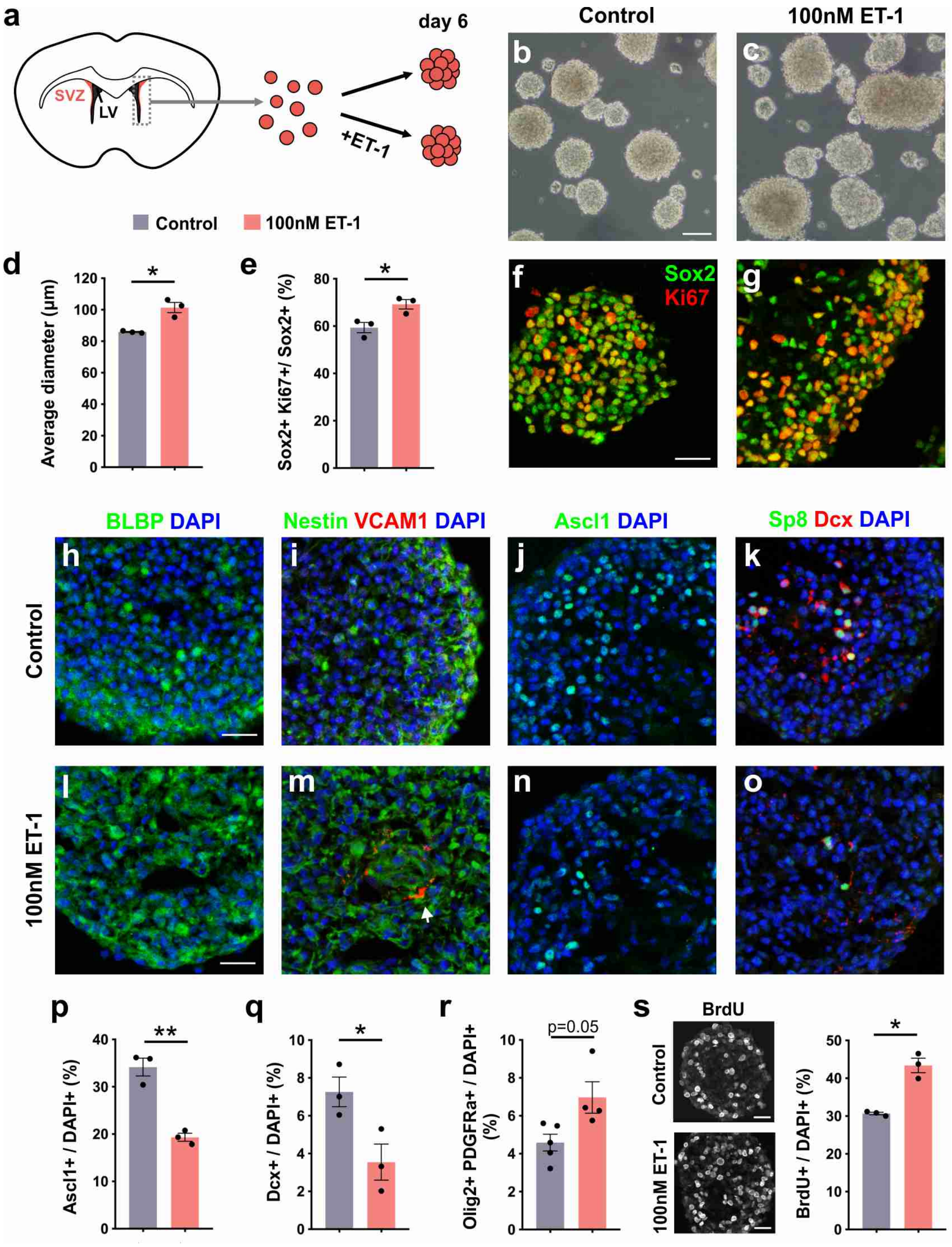

Supplementary Figure 4. ET-1 promotes RGC maintenance and blocks differentiation in neurospheres.

(**a**) Experimental strategy. (**b-c**) Representative images of control (b) and ET-1 treated (c) neurospheres 6 days after dissociation. Scale bar = 100µm. (**d**) Quantification of the average diameter of control and ET-1 treated neurospheres (n=3 independent batches). *p-value = 0.0399 (Welch's t test). (**e**) Quantification of the percentage of proliferating Sox2+ progenitors in control

and ET-1 treated neurospheres (n=3 independent batches). *p-value = 0.0288 (Welch's t test). (**f-o**) Sections of control (f, h-k) and ET-1 treated (g, l-o) neurospheres stained for makers of RGCs and neural progenitors. The arrow in m points to the presence of VCAM1+ cells. Scale bar = 25μm. (**p**) Quantification of the percentage of Ascl1+ cells in control and ET-1 treated neurospheres (n=3 independent batches). **p-value = 0.0071 (Welch's t test). (**q**) Quantification of the percentage of Dcx+ NPCs in control and ET-1 treated neurospheres (n=3 independent batches). *p-value = 0.0416 (Welch's t test). (**r**) Quantification of the percentage of OPCs in control and ET-1 treated neurospheres (n=5 control, 4 ET-1 treated batches). p-value = 0.0557 (Welch's t test). (**s**) BrdU was administered to neurospheres 20 minutes after the last ET-1 treatment and the cells were collected for analysis 4 hours later. Representative images of BrdU staining in control and ET-1 treated neurospheres. Scale bars = 25μm. Quantification of the percentage of BrdU+ cells in control and ET-1 treated neurospheres (n=3 independent batches). *p-value = 0.0194 (Welch's t test). Images are representative of 3 independent neurosphere batches. Data are presented as mean values +/- SEM. Source data are provided as a Source Data file.

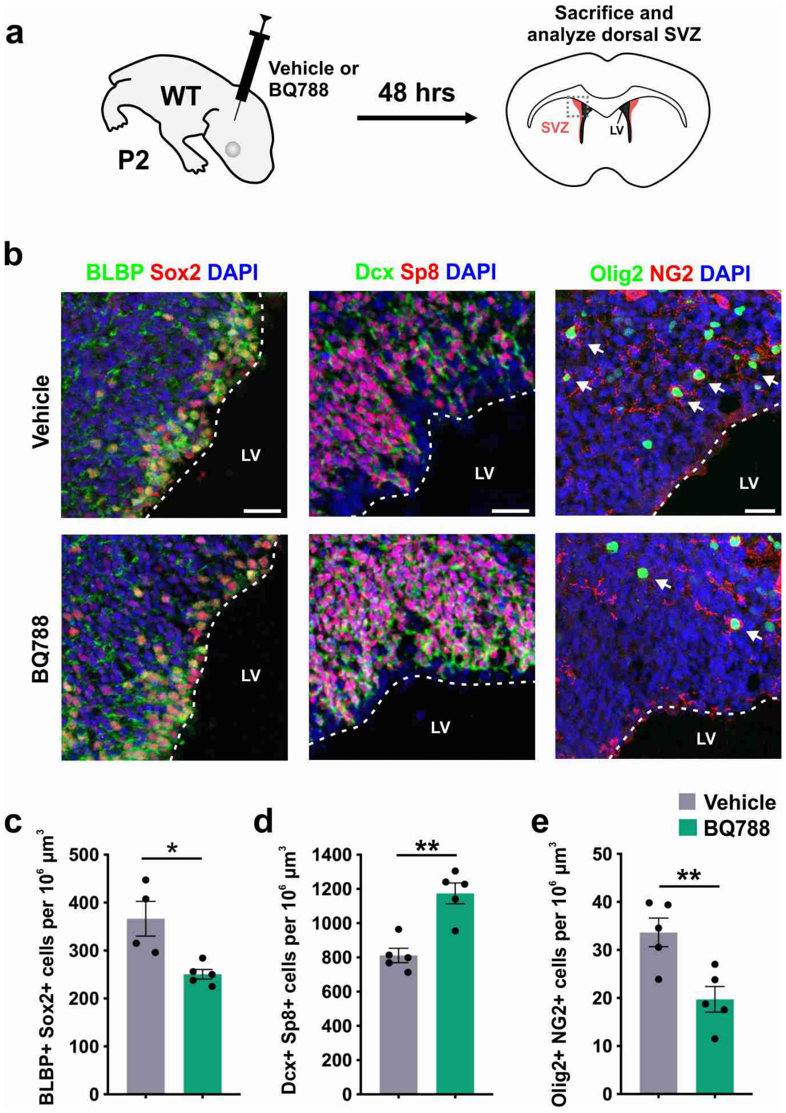

Supplementary Figure 5. Pharmacological inhibition of Ednrb receptor in the SVZ recapitulates the Ednrb cKO mouse phenotype.

(**a**) Experimental strategy. (**b**) Coronal images of the dorsal SVZ, labeling different progenitor populations in WT pups 48 hours after injection of vehicle or BQ788. LV = lateral ventricle. Arrows point to Olig2+ NG2+ OPCs. Scale bars = 25μm. (**c**) Quantification of the total number of BLBP+ Sox2+ RGCs in the dorsal SVZ, normalized to area (n=4 vehicle, 5 BQ788 injected mice). *p-value = 0.0449 (Welch's t test). (**d**) Quantification of the total number of Dcx+ Sp8+ NPCs in the dorsal SVZ, normalized to area (n=5 both groups). **p-value = 0.0016 (Welch's t test). (**e**) Quantification of the total number of Olig2+ NG2+ OPCs in the dorsal SVZ, normalized to area (n=5 both groups). **p-value = 0.0084 (Welch's t test). Data are presented as mean values +/- SEM. Source data are provided as a Source Data file.

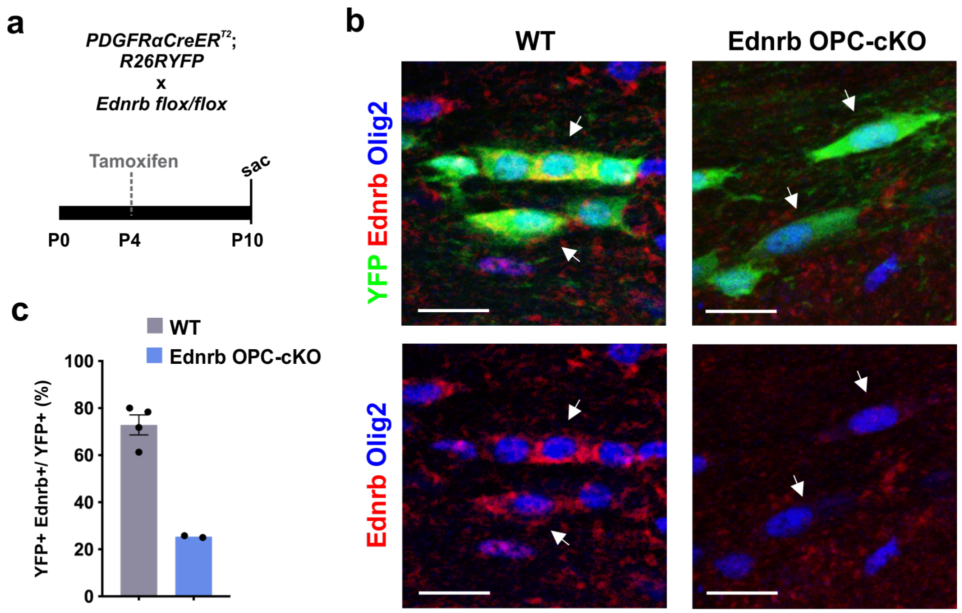

Supplementary Figure 6. Confirmation of Ednrb knockdown in OPCs.

(**a**) Experimental strategy for Ednrb ablation in OPCs. (**b**) Ednrb expression in YFP+ Olig2+ cells is reduced in Ednrb OPC-cKO mice, compared to WT controls. Scale bars = 25µm. (**c**) Quantification of the percentage of Ednrb+ YFP+ cells in the SVZ and CC at P10 (n=4 WT, 2 Ednrb OPC-cKO mice). Data are presented as mean values +/- SEM. Source data are provided as a Source Data file.

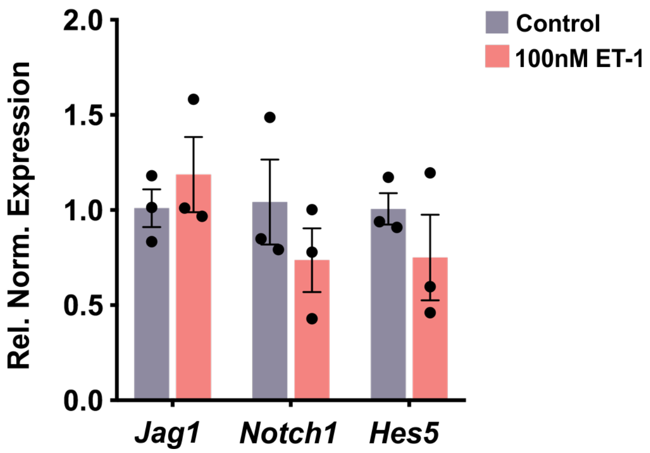

Supplementary Figure 7. Notch signaling is not activated in OPCs following ET-1 treatment.

QPCR for Notch pathway components 24 hours after ET-1 treatment of primary SVZ OPCs (n=3 independent batches). No significant differences ($p < 0.05$) were detected (Multiple t tests with Holm-Sidak multiple comparisons correction). Data are presented as mean values +/- SEM. Source data are provided as a Source Data file.

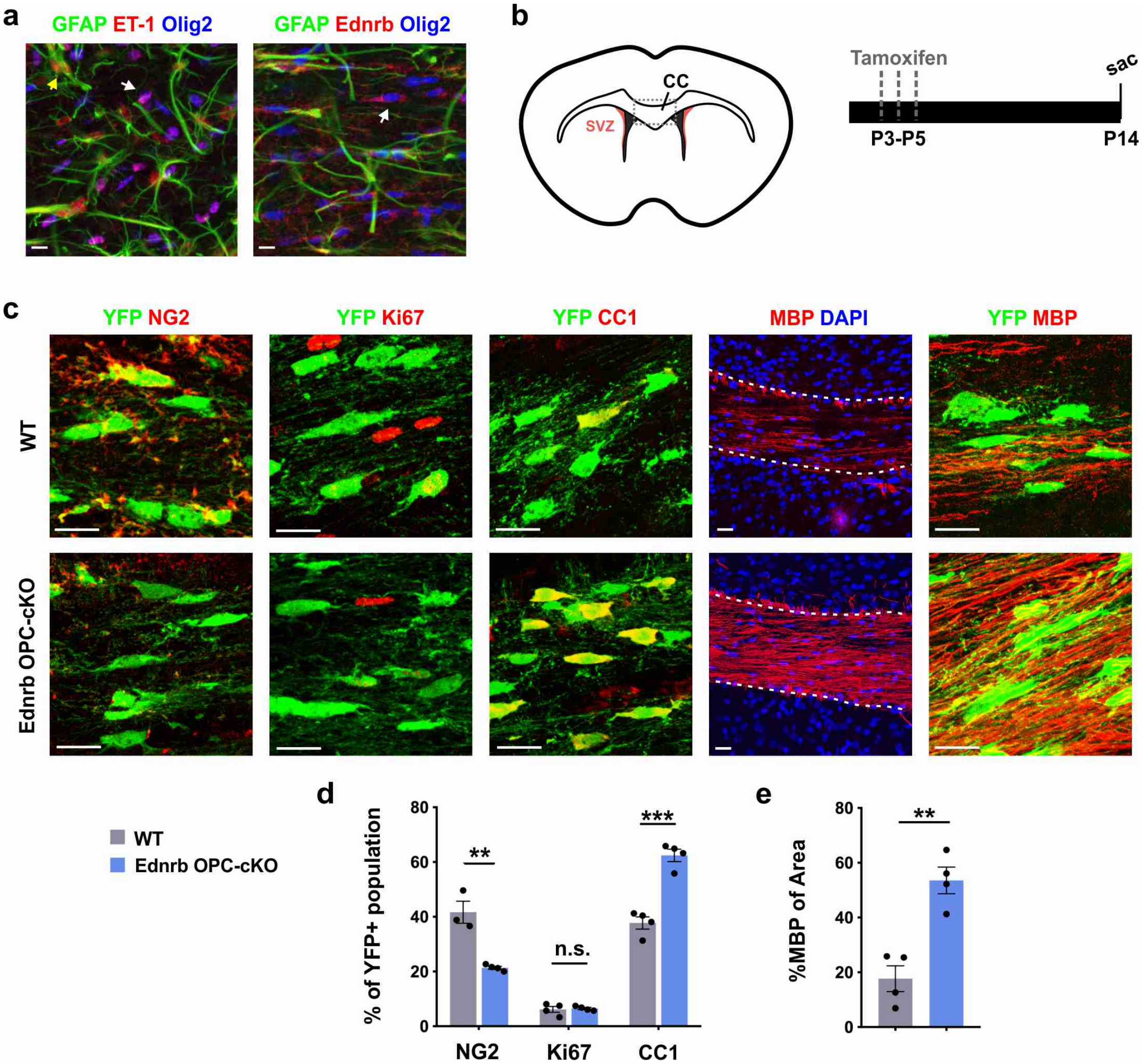

Supplementary Figure 8. OPC-specific Ednrb ablation promotes OL maturation in the CC. (**a**) Coronal sections of the CC in WT mice at P10. White arrows point to Olig2+ cells that express ET-1 or Ednrb. Yellow arrow points to ET-1+ GFAP+ astrocyte. Images are representative of 3 WT mice. (**b**) Experimental strategy. CC = corpus callosum. (**c**) Coronal sections of YFP+ recombined OL-lineage cells in the CC of WT and Ednrb OPC-cKO mice at P14. (**d**) Quantification of YFP+ OLs in the CC at P14 (NG2: n=3 WT, 4 Ednrb OPC-cKO mice. Ki67: n=4 mice both groups. CC1: n=4 mice both groups). **p-value = 0.003904; ***p-value = 0.000744; n.s. = not significant (Multiple t tests with Holm-Sidak multiple comparisons correction). (**e**) Quantification of the percentage of MBP+ pixels in the CC at P14 (n=4 mice both groups). **p-value = 0.0018 (Welch's t test). All scale bars = 25μm. Data are presented as mean values +/- SEM. Source data are provided as a Source Data file.

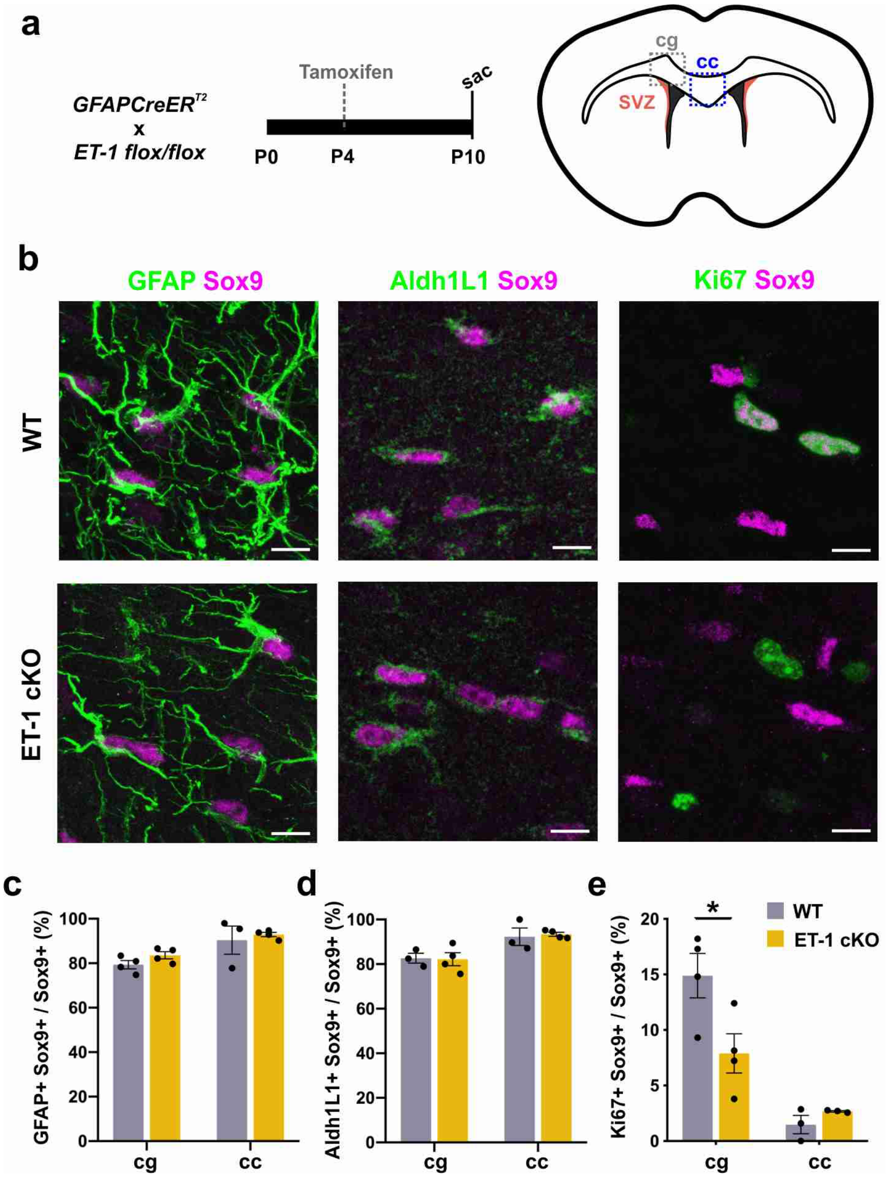

Supplementary Figure 9. ET-1 ablation reduces astrocyte proliferation in the subcortical white matter.

(**a**) Experimental strategy. Cg = cingulum. Cc = corpus callosum. (**b**) Coronal sections of the cg in WT and ET-1 cKO mice at P10. Scale bar = 10μm. (**c**) Quantification of the percentage of GFAP+ astrocytes in the cg and cc at P10 (cg: n=4 mice both groups. cc: n=3 WT, 4 ET-1 cKO mice). (**d**) Quantification of the percentage of Aldh1L1+ astrocytes in the cg and cc at P10 (n=3 WT, 4 ET-1 cKO mice). (**e**) Quantification of the percentage of proliferating astrocytes in the cg and cc at P10 (cg: n=4 mice both groups. cc: n=3 mice both groups). *p-value = 0.0337 (Two-way ANOVA with Tukey's multiple comparisons test). Data are presented as mean values +/- SEM. Source data are provided as a Source Data file.

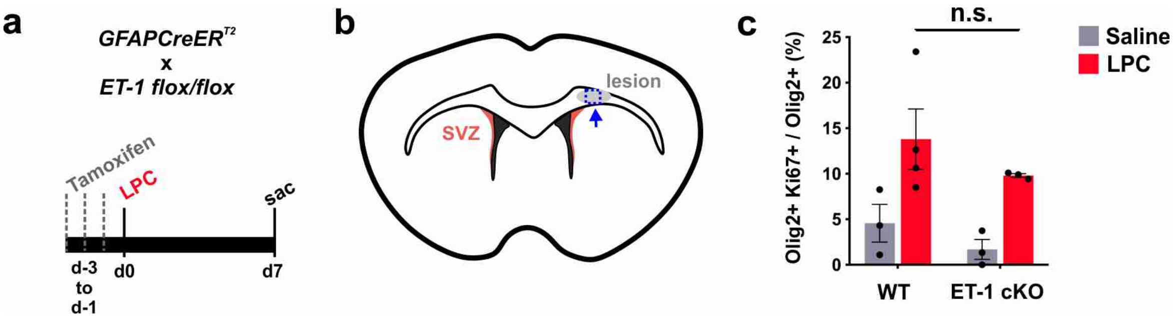

Supplementary Figure 10. ET-1 ablation does not alter OPC proliferation within subcortical white matter lesions.

(**a-b**) Experimental strategy. LPC = lysolecithin. Arrow in b points to the region quantified in c. (**c**) Quantification of the percentage of proliferating OPCs within the lesion of WT and ET-1 cKO mice at 7 days post LPC injection (Saline: n=3 mice both groups. LPC: n=4 WT, 3 ET-1 cKO mice). n.s. = not significant (Two-way ANOVA with Tukey’s multiple comparisons test). Data are presented as mean values +/- SEM. Source data are provided as a Source Data file.

Supplementary Table 1. Quantitative PCR Primers.

**Gene**	**Forward primer**	**Reverse primer**
Edn1	5’-CCCAAAGTACCATGCAGAAAAG-3’	5’-GATGCCTTGATGCTATTGCTG-3’
Ednrb	5’-CAATCCTCTGTATTTGGTGAG-3’	5’-CGTGATCGTTGGCTTTGAAC-3’
Gapdh	5’-CTTTGTCAAGCTCATTTCCTGG-3’	5’-TCTTGCTCAGTGTCCTTGC-3’
Hes5	5’-CAAGGAGAAAAACCGACTGCG-3’	5’-GCGAAGGCTTTGCTGTGTTT-3’
Jag1	5’-ACACCCGAACTGGACAAATAA-3’	5’-GTGCCCTCAAACTCTACCTATG-3’
Notch1	5’-GCAACTGTCCTCTGCCATATAC-3’	5’-GTCTTCAGACTCCTTGCATACC-3’

Supplementary Table 2. Primers for generating in situ probes.

Gene	Forward primer	Reverse primer
Edn1	5'-GAGAGCCAGGAGATTCCACA-3'	5'-GAGATTAACCCTCACTAAAGGGAGCATGGCAATGTTTCAGCTA-3'
Edn2	5'-GGGAGACCCCTATGCCTATC-3'	5'-GAGATTAACCCTCACTAAAGGGATCCCAAAAGTGTCCCAAGAG-3'
Edn3	5'-ACCAGGCTGGCTCTTTACAA-3'	5'-GAGATTAACCCTCACTAAAGGGACCAGACCAGTAGCCTTGAGC-3'
Ednrb	5'-TTCCCATTCTTAGCCCTGTG-3'	5'-GAGATTAACCCTCACTAAAGGGAAGATCTGGGGCGTCCTTTAT-3'
Ednra	5'-TGGGAGAAAGGAGATGATGG-3'	5'-GAGATTAACCCTCACTAAAGGGACCTGGCTTTTCCGAACTATG-3'
