# Supplementary material for: Endothelin-1 signaling maintains glial progenitor proliferation in the postnatal subventricular zone
Source: Nat Commun. 2020 May 1;11:2138. doi: 10.1038/s41467-020-16028-8 (PMC7195367; doi:10.1038/s41467-020-16028-8)
Supplement: Supplementary file 3 — Reporting Summary [file 41467_2020_16028_MOESM3_ESM.pdf]

nature research

Corresponding author(s): Vittorio Gallo

Last updated by author(s): Mar 30, 2020

Reporting Summary

Nature Research wishes to improve the reproducibility of the work that we publish. This form provides structure for consistency and transparency in reporting. For further information on Nature Research policies, see Authors & Referees and the Editorial Policy Checklist .

Statistics

For all statistical analyses, confirm that the following items are present in the figure legend, table legend, main text, or Methods section.

n/a	Confirmed	
☐	☒	The exact sample size (*n*) for each experimental group/condition, given as a discrete number and unit of measurement
☐	☒	A statement on whether measurements were taken from distinct samples or whether the same sample was measured repeatedly
☐	☒	The statistical test(s) used AND whether they are one- or two-sided<br>*Only common tests should be described solely by name; describe more complex techniques in the Methods section.*
☒	☐	A description of all covariates tested
☐	☒	A description of any assumptions or corrections, such as tests of normality and adjustment for multiple comparisons
☐	☒	A full description of the statistical parameters including central tendency (e.g. means) or other basic estimates (e.g. regression coefficient) AND variation (e.g. standard deviation) or associated estimates of uncertainty (e.g. confidence intervals)
☐	☒	For null hypothesis testing, the test statistic (e.g. *F*, *t*, *r*) with confidence intervals, effect sizes, degrees of freedom and *P* value noted<br>*Give P values as exact values whenever suitable.*
☒	☐	For Bayesian analysis, information on the choice of priors and Markov chain Monte Carlo settings
☒	☐	For hierarchical and complex designs, identification of the appropriate level for tests and full reporting of outcomes
☒	☐	Estimates of effect sizes (e.g. Cohen's *d*, Pearson's *r*), indicating how they were calculated

Our web collection on statistics for biologists contains articles on many of the points above.

Software and code

Policy information about availability of computer code

Data collection	Immunohistochemical data was collected using Zeiss LSM or Zen 2.3 software. RNA-sequencing was performed on Novaseq 6000 (Illumina).
Data analysis	Images were viewed and analyzed using FIJI ImageJ software (version 2.0.0, NIH). Data was analyzed using Microsoft Excel (version 16.16.4) and Graphpad/Prism (version 7). RNAseq data was analyzed using Ingenuity Pathway Analysis (version 1-16, Qiagen), HISAT2 (version 2.1.0), SAMtools (version1.3), Picard (version 2.17.6) MarkDuplicates, featureCounts (subread version 1.6.2), and DESeq2 (version 1.20). Figures were prepared using CorelDraw 2018 (Version 20.1.0.708).

For manuscripts utilizing custom algorithms or software that are central to the research but not yet described in published literature, software must be made available to editors/reviewers. We strongly encourage code deposition in a community repository (e.g. GitHub). See the Nature Research guidelines for submitting code & software for further information.

Data

Policy information about availability of data

All manuscripts must include a data availability statement. This statement should provide the following information, where applicable:

- Accession codes, unique identifiers, or web links for publicly available datasets
- A list of figures that have associated raw data
- A description of any restrictions on data availability

All RNA-sequencing data has deposited in the NCBI Sequence Read Archive (accession code: PRJNA607509). The authors declare that all data supporting the findings of this study are available within the paper and its supplementary information files. Source data for Figures 1-8 and Supplementary Figures 1-10 are provided as a Source Data file.

Field-specific reporting

Please select the one below that is the best fit for your research. If you are not sure, read the appropriate sections before making your selection.

☒ Life sciences ☐ Behavioural & social sciences ☐ Ecological, evolutionary & environmental sciences

For a reference copy of the document with all sections, see nature.com/documents/nr-reporting-summary-flat.pdf

Life sciences study design

All studies must disclose on these points even when the disclosure is negative.

Sample size	This sample sizes for these experiments were based on similar experiments done in the lab (Chew et al 2019; Jablonska et al. 2016). The sample size of all experiments in this study was a minimum of 3 animals, as is standard in the field. This sample size was sufficient to detect significant differences among experimental and control animals.
Data exclusions	No data were excluded.
Replication	All experiments were replicated on at least 3 animals or cell preparations, collected across many days. The primary cellular and molecular findings were successfully replicated in both in vivo mouse studies and in vitro cell culture assays. All attempts at replication were successful.
Randomization	Primary cells (neurospheres and OPCs) and organotypic brain slices were randomly treated with ET-1. Because specific genotypes were required for in vivo mouse studies, mice could not be randomly allocated.
Blinding	Mouse tissue was collected, stained, and imaged blind. For primary cell culture and RNAseq experiments, blinding was not performed as researchers needed to be unblinded to perform the correct comparisons.

Reporting for specific materials, systems and methods

We require information from authors about some types of materials, experimental systems and methods used in many studies. Here, indicate whether each material, system or method listed is relevant to your study. If you are not sure if a list item applies to your research, read the appropriate section before selecting a response.

Materials & experimental systems

n/a	Involved in the study	
☐	☒	Antibodies
☒	☐	Eukaryotic cell lines
☒	☐	Palaeontology
☐	☒	Animals and other organisms
☐	☒	Human research participants
☒	☐	Clinical data

Methods

n/a	Involved in the study	
☒	☐	ChIP-seq
☒	☐	Flow cytometry
☒	☐	MRI-based neuroimaging

Antibodies

Antibodies used	The following primary antibodies were used for staining mouse tissue and cells: rabbit anti-ET-1 (Abcam ab117757, 1:500), rabbit anti-Ednrb (Abcam ab117529, 1:500), rabbit anti-Ednra (Abcam ab117521, 1:500), rabbit anti-Olig2 (Millipore AB9610, 1:500), guinea pig anti-Olig2 (gift from Bennett Novitch), chicken anti-GFAP (Abcam ab4674 1:500), rabbit anti-S100β (Abcam ab868, 1:500), rabbit anti-BLBP (Millipore ABN14, 1:300), goat anti-Sox2 (Santa Cruz sc-17320, 1:100), guinea-pig anti-Dcx (Millipore AB2253, 1:500), rabbit anti-NG2 (Millipore AB5320, 1:200), rat anti-BrdU (Abcam ab6326, 1:250), rabbit anti-Ki67 (Abcam ab16667, 1:200), rabbit anti-Ki67 (Vector labs VP-K451, 1:200), chicken anti-GFP (Aves labs GFP-1010, 1:1000), rabbit anti-Pax6 (Millipore AB2237, 1:500), rabbit anti-Gsx2 (Millipore ABN162, 1:300), goat anti-Sp8 (Santa Cruz sc-104661, 1:100), mouse anti-CC1/APC (Calbiochem OP80, 1:250), mouse anti-MBP (Covance SMI-99P, 1:500), rat anti-VCAM1 (BD Pharmingen 550547, 1:300), mouse anti-βcatenin (BD Biosciences 610154, 1:500), rabbit anti-cleaved Caspase3 (Cell Signaling 9664S, 1:200), mouse anti-NeuN (Millipore MAB377, 1:500), mouse anti-Mash1 (BD Pharmingen 556604, 1:200), chicken anti-Nestin (Aves labs NES, 1:500), rat anti-CD31 (BD Pharmingen 550274, 1:50), goat anti-Sox9 (R&D Systems AF3075, 1:500), rat anti-PDGFRa (BD Pharmingen 558774, 1:250) and rabbit anti-Aldh1L1 (Abcam ab87117, 1:250). The following secondary antibodies (all from Jackson Immunoresearch) were used on mouse tissue/cells at a dilution of 1:500: donkey anti-chicken Alexa488 (703-545-155), donkey anti-mouse Alexa488 (715-545-151), donkey anti-rabbit Alexa488 (611-545-215), donkey anti-mouse Alexa594 (715-585-151), donkey anti-goat Alexa594 (127-585-160), donkey anti-rat Alexa594 (712-585-153), donkey anti-rabbit Alexa594 (611-585-215), donkey anti-rabbit Alexa647 (611-605-215), donkey anti-goat Alexa647 (127-605-160), and donkey anti-guinea pig Alexa647 (706-605-148). The following primary antibodies were used for staining human tissue: anti-GFAP (Dako Z0334, 1:1000), anti-PDGFRa (Santa Cruz sc-12911), and anti-ET1 antibody (Meridan H54085m). The following secondary antibodies were used for human tissue: Envision mouse/rabbit HRP (Dako K406311-2) and Envision rabbit/mouse DAB/Permanent Red (Dako K536111-2). For in situ hybridization, the anti-DIG AP Fab fragment (Roche Life Science 11093274910) was used.

Validation	All primary antibodies have been validated for immunohistochemistry for use on mouse or human. More information, including citations, can be found on manufactures websites.

Animals and other organisms

Policy information about studies involving animals; ARRIVE guidelines recommended for reporting animal research

Laboratory animals	Species: mouse (Mus musculus). Strains: NestinCreERT2 (Jackson ID#016261), B6;129-Ednrb tm1.1Nat/J (Jackson ID# 011080), PdgfraCreERT2 (Jackson ID#018280), B6.129X1-Gt(ROSA)26Sortm1(EYFP)Cos/J (Jackson ID#006148), C57BL/6J (Jackson ID#000664), B6.Cg-Tg(GFAP-cre/ERT2)505Fmv/J (Jackson ID#012849), and Endothelin-1 floxed mice obtained from Dr. Ralph Shohet at the University of Hawaii. Sex: both males and females were used. Age: Mice were sacrificed at P2-P14 for early postnatal analysis. Adults (8-12weeks) were used for lysolecithin-induced demyelination experiments.
Wild animals	The study did not involve wild animals.
Field-collected samples	The study did not involve samples collected from the field.
Ethics oversight	All animals procedures were approved by the Institutional Animal Care and Use Committee (IACUC) of the Children's National Health System (protocol #30578).

Note that full information on the approval of the study protocol must also be provided in the manuscript.

Human research participants

Policy information about studies involving human research participants

Population characteristics	Human tissue samples were obtained from 68 and 75 year old females with a diagnosis of CARASAL and found to have a c.973C.T point mutation in the CTSA gene. Patients were on anti-hypertensive drugs.
Recruitment	Patients were recruited clinically, they were evaluated for a leukodystrophy at the Amsterdam Leukodystrophy Center.
Ethics oversight	The study of these patients is approved by the Medical Ethical Committee of the Free University Medical Center, Amsterdam, the Netherlands.

Note that full information on the approval of the study protocol must also be provided in the manuscript.
